# A rare case of collision tumour of the ovary complicated by torsion

**DOI:** 10.1259/bjrcr.20210114

**Published:** 2022-03-09

**Authors:** Dhanya Jacob, Thara Pratap, Muhammed Jasim Abdul Jalal, Pushpa Mahadevan, Vishnu A K

**Affiliations:** 1 Department of Radiology, VPS Lakeshore Hospital, Kochi, Kerala, India; 2 Department of Internal Medicine and Rheumatology, VPS Lakeshore Hospital, Kochi, Kerala, India; 3 Department of Pathology, VPS Lakeshore Hospital, Kochi, Kerala, India

## Abstract

Collision tumour is the coexistence of two adjacent, but histologically distinct tumours without histologic admixture. Collision tumours are rare in the ovary. It is mostly a histopathological diagnosis often missed in preoperative imaging. The radiologist, gynaecologists and pathologists should be aware of such a combination of tumours to avoid misdiagnosis. We describe the finding of a rare collision tumour, mature cystic teratoma and ovarian fibroma complicated by torsion.

## Introduction

Collision tumours are histologically two distinct neoplasms that coexist in the same organ without any histological admixture. Although very rare, they have been found to affect a range of organs, including the gastrointestinal tract (oesophagus, stomach, colon), lung, skin, adrenals, central nervous system, lymph nodes and uterus, but are relatively rare in ovary.^1,2^ Teratoma is one of the most common components of collision combination in the ovary. Although there are few case reports of ovarian collision, the majority are a combination of surface epithelial cell and granulosa cell tumours. Combinations of sex-cord stromal tumour and germcell tumours are extremely rare, this case being the second in the list.^3^ Here, we are reporting our findings in such a collision tumour with secondary torsion of the ovarian pedicle.

## Case presentation

A 40-year-old nullipara presented with complaints of abdominal pain and fullness of the lower abdomen and vomiting for a week. On physical examination, her vitals were stable. On abdominal examination, there was a large solid mass arising from the pelvis reaching above the umbilicus, 24-week size with restricted mobility. Per vaginal examination showed a healthy cervix. Uterus was enlarged with restricted mobility and an adnexal mass could be detected on the right side.

## Investigations

**Figure 1.**
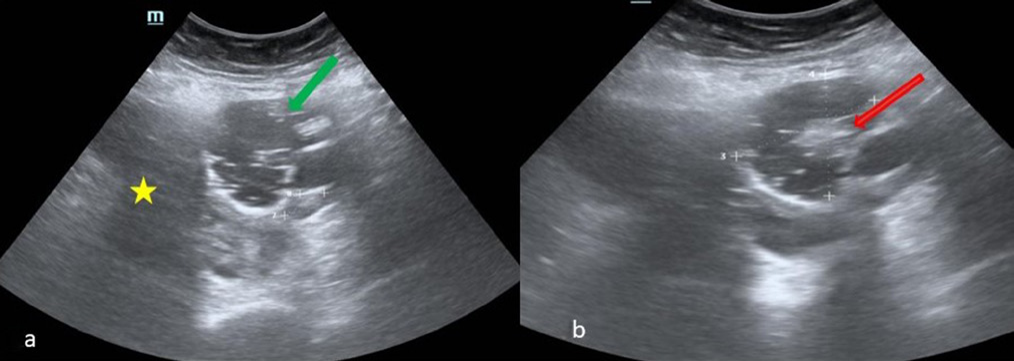
(**a, b**): Transabdominal ultrasound shows the dermoid component with cystic areas, echogenic thick walls and linear hyperechoic area (green arrow). Hyperechoic area within the cystic area (red arrow in 1b) corresponds to the dermoid plug/rokitansky protuberance. The hypoechoic area superiorly denotes a solid lesion with lobulated margin (yellow asterix).

USS showed (Figure 1) large heterogeneous predominantly solid mass in the midline and on the right side measuring up to 13.2 × 8.3 cm. Septated cystic component of size 5.3 × 5.1 cm was seen in the lower part of mass. There were associated minimal ascites. Suspecting ovarian neoplasm, blood investigations were done.

- Ca125 level was 404.1 U ml^−1^(normal range: 0–25 U ml^−1^);
- Carcinoembryonic antigen (CEA) was 0.5 (normal);
- Ca19.9 was 16.3(normal range: 0–37 U ml^−1^);
- Lactatedehydrogenase(LDH)was 405 U ml^−1^(elevated);
- AFP was <30 ng ml^−1^.

**Figure 2.**
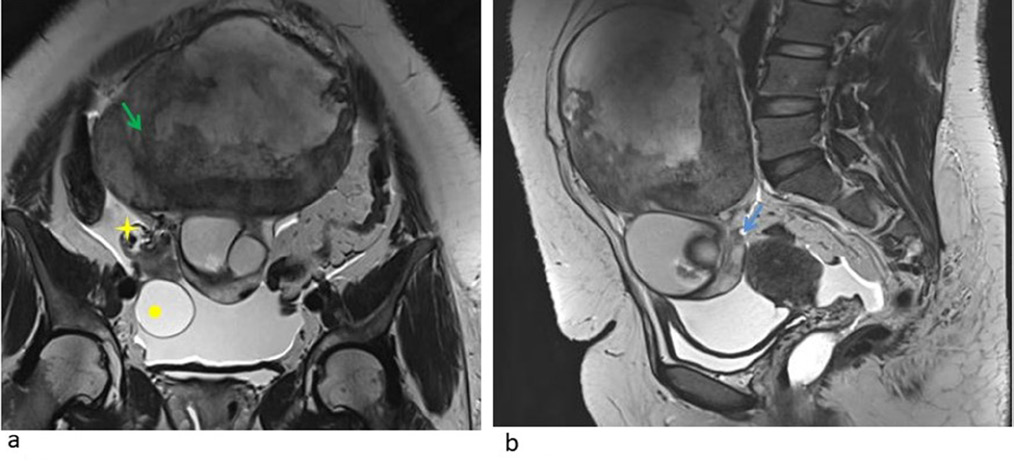
(**a, b**): Coronal T2W image showing dermoid component in the inferior aspect with heterogeneous lobulated mass in the superior aspect with central necrosis (arrow). Solid lesion shows T2 intermediate components in the periphery. Images also show the twisted right ovarian pedicle (yellow asterix) and a para ovarian cyst (yellow circle). T2W sagittal section through the pelvis shows the compressed right ovarian parenchyma with a small follicle (Blue arrow).

**Figure 3.**
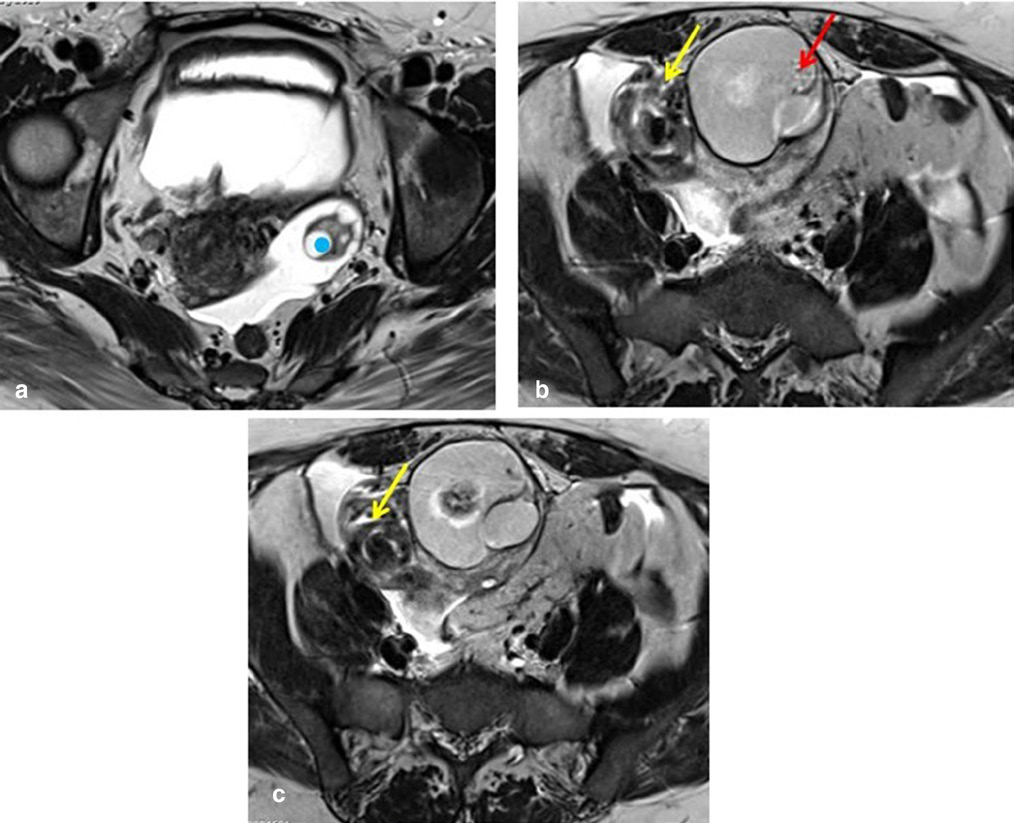
(**A, B, C**): Axial T2 MRI. Figure A shows the normal left ovary (blue dot). Figures B and C show a twisted right ovarian pedicle (yellow arrow) with a medialised right adnexal dermoid cyst (red arrow).

**Figure 4.**
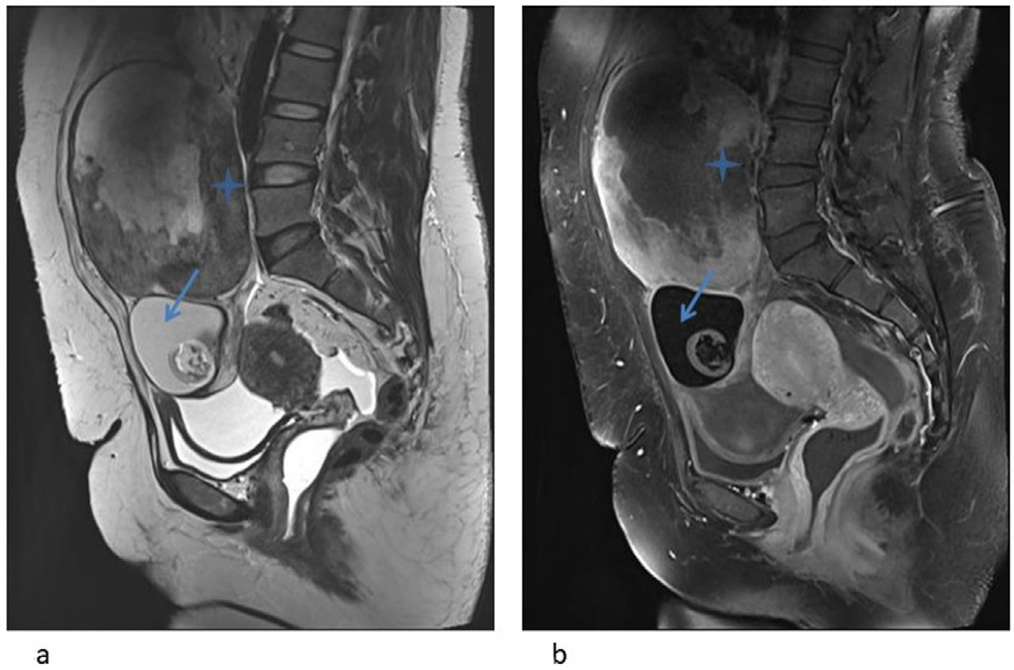
(**a, b**): Sag MRI - T2 and T1FS post-contrast images showing moderate-sized right adnexal mass extending to the lower abdomen. Blue arrows denote the dermoid component. Solid component with central necrotic area is seen in the superior aspect of the mass (asterix). Post-contrast study shows peripheral enhancement.

**Figure 5.**
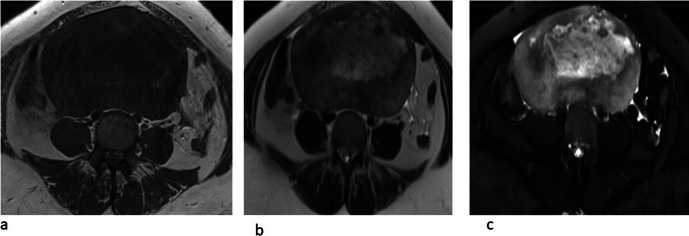
(**a, b, c**): Axial T1 (5a), T2(5b), and T2FS (5c) MRI images showing the fibromatous component of the collision tumour with central necrosis and T1 and T2 intermediate signals in the periphery.

**Figure 6.**
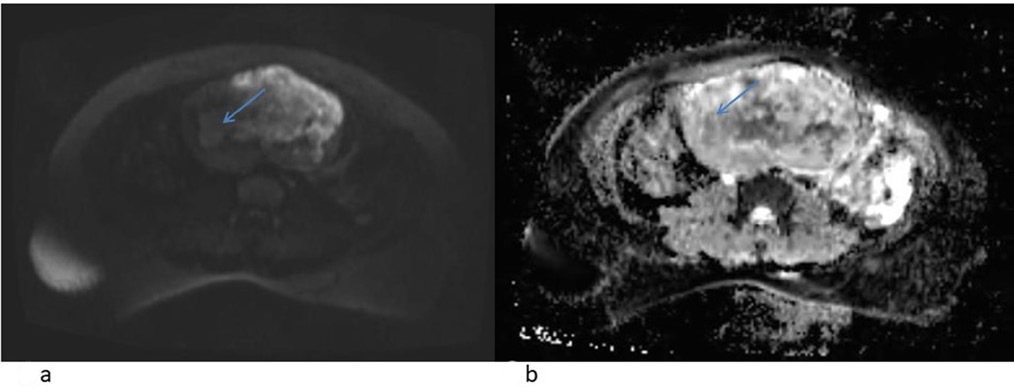
(**a, b**): DWI with ADC images showing mild diffusion restricting areas within the lesion.

**Figure 7.**
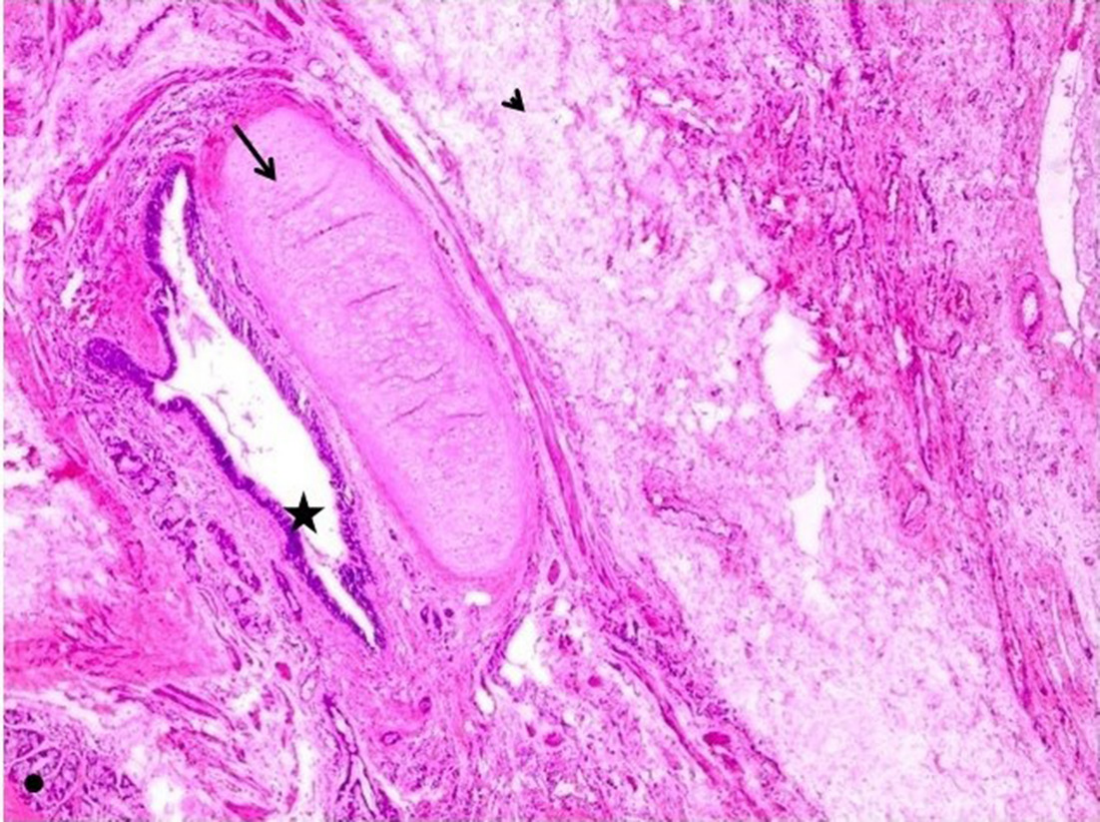
Axial (**a, b, c**) and coronal (**d**) – MRI images show heterogeneous enhancement in the periphery of the lesion. Figure d shows both the fibromatous component (arrow) and dermoid component (asterix) of the collision tumour.

**Figure 8.**
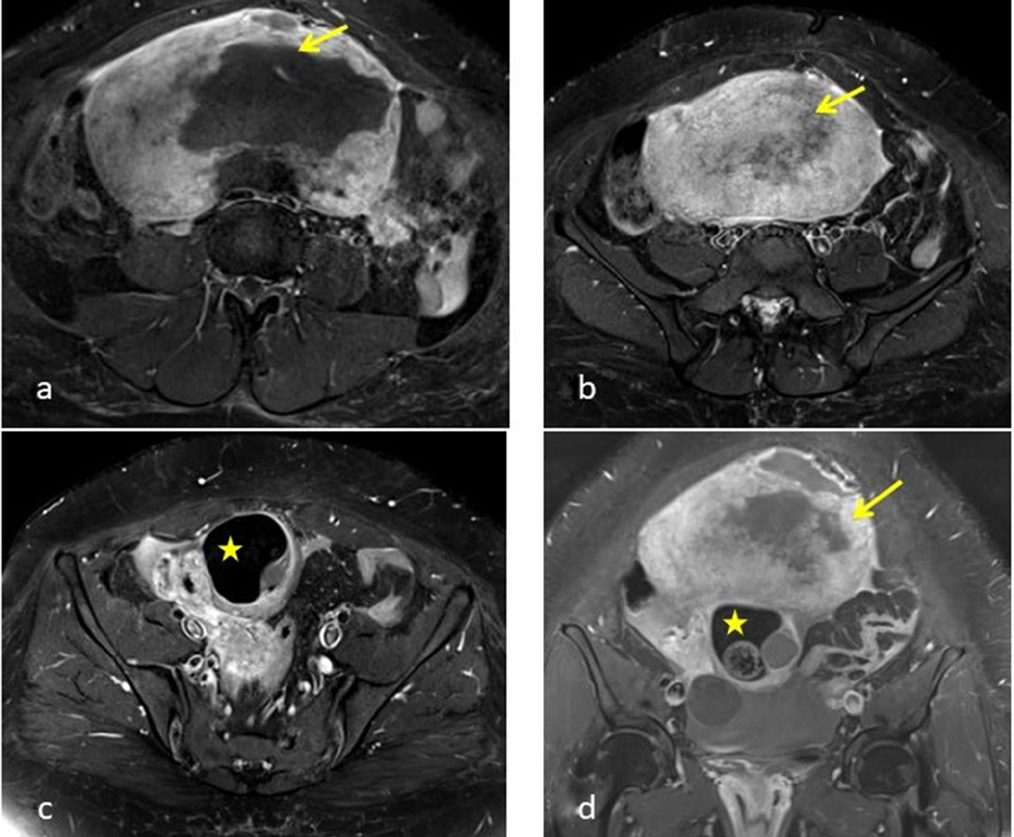
(**a, b**): MRI Axial T1 and T1 FS showing fat suppression within the dermoid (blue arrow). Heterogeneous area in the centre of the dermoid (yellow triangle) corresponding to the dermoid plug-in ultrasound also shows small areas of fat contents. Another locule with T1 intermediate signal contents is also noted (red arrow).

To further characterize the lesion, cross-sectional imaging was done. Magnetic resonance imaging showed a large heterogeneous lesion15.8 (TR) X 9.4 (AP) X13(CC) with T2 intermediate signal in the periphery and central cystic areas with haemorrhagic contents (Figures 2–5). T2 hypointense capsule was demonstrated. The solid periphery of the lesion showed scattered areas of diffusion restriction (Figures 6 and 7) with heterogeneous enhancement in postcontrast study. Complex cystic lesion (Figure 8) was seen in the inferior aspect of this tumour measuring 7.0 × 6.6 cm. This lesion was heterogeneous with T1 hyperintense contents which was getting suppressed in the fat-suppressed sequence suggestive of dermoidcyst. Heterogeneous area in the centre of the dermoid corresponding to the dermoid plug on ultrasound was seen. The main feeder to the lesion was from the ovarian pedicle. Enhancing ovarian parenchyma was seen along the periphery of the lesion with tiny follicles (Figure 2).

There was thickening and twisting of the right adnexal structures and ovarian pedicle, seen in the midline superior to the uterus (Figure 3).

Another small cystic lesion was seen in the right adnexa inferior to the dermoid suggestive of para-ovarian cyst measuring 4.3 × 3.4 cm(Figure 2). Left ovary was normal in size with an irregular follicle (Figure 3). Free fluid in the abdomen and pelvis showed T1 hyperintense haemorrhagic areas – probably secondary to torsion.

Uterus is normal in size and shape with a small 7 mm fibroid in the anterior wall. Endometrial thickness was normal.

## Differential diagnosis

1. A pedunculated fibroid with degeneration
2. A solid ovarian neoplasm.

### Treatment and follow-up

Total abdominal hysterectomy with bilateral salpingo–oophorectomy were done. Preoperatively, the right ovary was enlarged and was twisted five times in its pedicle. Frozen section showed mature cystic teratoma and spindle cell tumour. On gross examination, the right adnexalovarian mass measured 22 × 17 × 9 cm, weighing 1.4 kg. Cut section showed predominantly solid, firm trabeculated whitish growth measuring 16 × 14 × 6 cm with extensive haemorrhage and focal necrosis. There was a multiloculated cystic area measuring 6 × 3 cm containing hair and pultaceous material in the inferior aspect.

Sections from the solid firm trabeculated ovarian tumour show congestion and haemorrhagic infarction. Spindle cells were arranged in vague fascicles, storiform areas admixed with blood vessels. The spindle cells have pale cytoplasm with indistinct cytoplasmic borders with round to oval nuclei with IHC showing spindle cells negative for inhibin and SMA. The spindle cells are negative for Calretinin, desmin, CD34 and S100.

The multiloculated cystic part of the ovarian tumour shows a teratoma with mature elements of different germ layers, namely, hair-bearing skin with appendages, respiratory epithelium, sero-mucinous glands, glial tissue, choroid, smooth muscle, adipose tissue, cartilage, bone and blood vessels.

**Figure 9.**
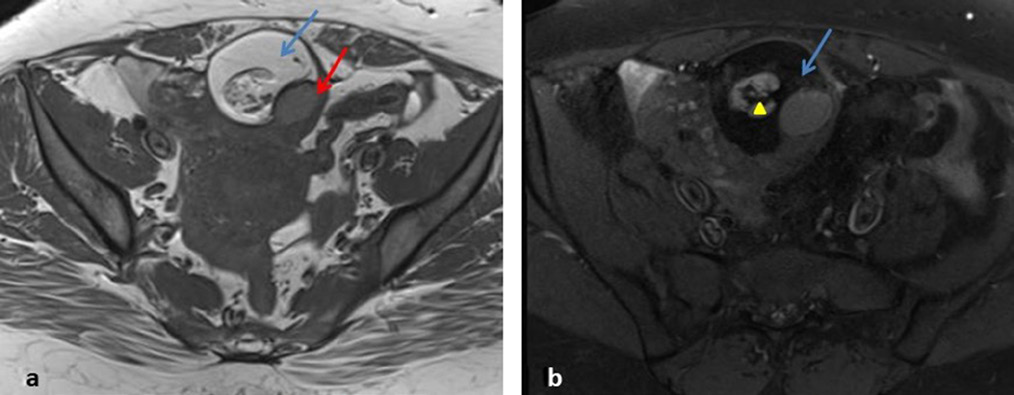
Low power H&E stain of the tumour showing mature cystic teratoma with fat (arrow head) and cartilage (arrow), bowel epithelium (dot), and cysts lined by pseudostratified epithelium (asterix).

**Figure 10.**
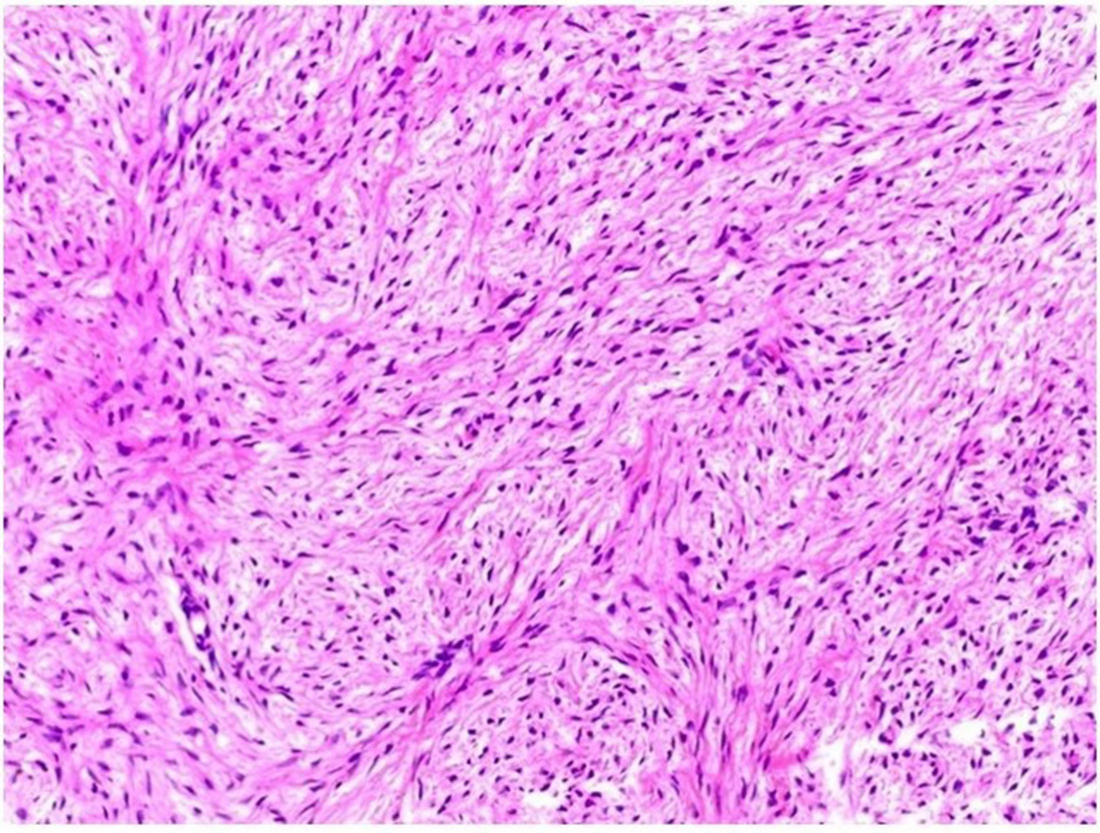
Low power H&E staining of the tumour showing fibroma with sheets and fascicles of spindle cells with uniform nucleus and indistinct cytoplasmic borders.

Histopathology and IHC were consistent with mature cystic teratoma with fibroma of the right ovary with haemorrhagic infarction (Figures 9 and 10)

## Discussion

Collision tumour (also known as encounter tumour) is defined as two or more primary tumours from different tissues occurring at the same anatomical site. Tumours exist independently of each other, and their biological behaviour depends on their individual tumour characteristics.

Collision tumours, which occur in the testis, skin, adrenal glands, pancreas, etc., have been reported in different forms, but collision tumours occurring in the ovary are extremely rare. The pathogenesis of collision tumour is unknown, the accidental development of two different tumours is the simplest theory. Few other hypotheses are the common origin of pluripotent stem cells, simultaneous proliferation of two different cell lines, as common carcinogenic agent interacting with different tissues and inducing different tumours, and tumour growth promotion by microenvironmental changes induced by primary lesions such as oncogenic growth factor production, neoangiogenesis and inflammation.^4^

In the reported cases of ovarian collision, the most common combinations are epithelial and germ cell tumours, followed by germ cell tumours and sex-cord-stromal tumour.^5^ Mature cystic teratoma is one of the most common components in a collision tumour and the most common collision is teratoma and mucinous tumours. Most collision tumours are diagnosed postoperatively, it may be possible to identify the collision tumours by CT/MRI imaging. The possibility of a collision tumour should be considered when an ovarian teratoma has imaging findings that cannot be explained solely by an ovarian teratoma and when two or three types of typical imaging findings of different tumours are present in the same ovary, particularly when one tumour lies inside or on the wall of another tumour.^6^ The specific imaging presentations of an ovarian collision tumour largely depend on its composition. The main imaging characteristics of ovarian collision tumours are the presence of a large solid cystic mass with different attenuation or intensities; a germ cell or sex-cordstromal component that is often smaller than the epithelial component, which can lie inside (‘‘the nested tumor’’) or on the wall (‘‘back-to-back’’) of the latter component with a clear margin and finally coexistence of imaging features specific to the different collision tumour components.^7^

Sex-cord stromal tumours are 8% of all ovarian neoplasms. Fibroma is one of the sex cordstromal tumours, constituting for approximately 4% of all ovarian neoplasms.^6^ Ovarian fibroma and fibrothecomas are relatively common incidental solid ovarian tumours, and the ability to make a diagnosis of this benign tumour on imaging can greatly affect patient management, especially in terms of avoiding unnecessary surgery, decreasing patient anxiety and avoiding morbidity associated with invasive surgical procedures. Pathologically, fibromas are composed of whorled fascicles of cytologically bland spindle cells embedded in a collagenous stroma. Imaging is important to differentiate fibromas and fibrothecomas from fibroids, and from malignant ovarian tumours, especially when these present as a solid ovarian mass associated with ascites and pleural effusion. Ultrasound features of fibromas and fibrothecomas are often nonspecific. MRI is often needed for further differentiation of ovarian fibromas and fibrothecomas from other solid ovarian masses, especially pedunculated or broad ligament leiomyomas. MRI is an excellent imaging modality for the detection and characterisation of ovarian fibromas and fibrothecomas.^8^

Most ovarian fibromas and fibrothecomas are isointense to hypointense to the uterine myometrium on T1- and T2-weighted images. MRI features of fibromas and fibrothecomas depend on the size of the lesion. The presence of pseudocapsules, degenerative changes, peripheral subcapsular cystic areas, heterogeneous T2 signal and heterogeneous enhancement is more common in larger fibromas and fibrothecomas. Peripheral small cysts can be seen, indicating an ovarian stroma stretching around the fibroma and fibrothecoma, and indicate the ovarian origin of the lesion, thus helping in differentiation from fibroids. Fibromas and fibrothecomas enhance significantly less than uterine myometrium and fibroids. To distinguish ovarian mass from peduncledmyomas, demonstrating the vascular bridging sign or vascular pedicle between the uterus and peri-uterine mass may be helpful.^9^

Ascites is detected in association with 10 to 15% of ovarian fibromas exceeding a diameter of 10 cm.^10^ Fibromas are predominantly solid. Cystic areas, if present, are usually small and are without multiloculation. Some fibromas undergo prominent cystic degeneration and may be mistaken as surface epithelial tumours. However, the cyst secondary to degeneration does not have an epithelial lining.

Mature cystic teratoma is a commonly encountered ovarian tumour, constituting 20% of all ovarian tumours in adults and 50% of all ovarian tumours in children.^11^ Mature cystic teratomas are composed of well-differentiated derivations of the three germ cell layers (ectoderm, mesoderm and endoderm).

It can have bones or teeth which are located within the Rokitanskynodule. Ultrasonography findings in mature cystic teratomas vary from a cystic lesion with a densely echogenic tubercle (Rokitansky nodule) projecting into the cyst lumen, to a diffusely or partially echogenic mass with the echogenic area usually demonstrating attenuation owing to sebaceous material and hair within the cyst cavity, to multiple thin, echogenic bands caused by hair in the cyst cavity. They are easily diagnosed on imaging studies because of their characteristic intratumoral fat component. At CT, fat attenuation within a cyst (negative attenuation), with or without calcification in the wall, is diagnostic for mature cystic teratoma.^12^On MR imaging, intratumoral fat can be diagnosed with the combination of T1-weighted imaging and fat-saturated T1-weighted imaging; intratumoral fat shows high signal intensity on *T*_1_-weighted images but a signal drop on fat-saturated *T*_1_-weighted images. Chemical-selective fat-saturated T1-weighted imaging is mandatory for the diagnosis of teratomas because other conditions, such as haemorrhage or a high concentration of protein, can also cause T1 shortening.^13^

## Learning points

1. Our case showed two different tumour components with different MR characteristics and in close proximity with superadded torsion. Dermoid component was diagnosed without difficulty in view of its characteristic fat component. It was difficult to characterise the solid tumour in view of torsion.
2. Differential diagnosis was between a pedunculated fibroid with degeneration and a solid ovarian neoplasm. Ovarian origin was preferred rather than uterine in view of the bridging vascular pedicle sign. Torsion was evidenced by the twisting of the ovarian pedicle and T1 hyperintense contents were presumed to be due to haemorrhage.
3. A collision tumour was suspected, however, a preoperative diagnosis of the type of co-existing tumour was not made with certainty due to altered signal characteristics secondary to torsion. However, the recognition of different imaging characteristics within a tumour should enable the radiologist to raise the possibility of a collision tumour.
4. Specific MR characteristics of the components may help in delineating benign components like fibroma from more common and aggressive epithelial tumours, thereby reducing the anxiety of the patient and avoiding unnecessary extensive surgery.

## References

[1] Choudhary S, Adisesha S. Collision tumors of ovary: a rare phenomenon. International Journal of Case Reports and Images 2012; 3: 68–70.

[2] Singh AK, Singh M. Collision tumours of ovary: a very rare case series. J Clin Diagn Res 2014; 8: FD14–16. doi: 10.7860/JCDR/2014/11138.5222 25584236 PMC4290255

[3] Borges A, Loddo A, Martins A, Peiretti M, Fanni D, Djokovic D. Collision tumors in ovary: case series and literature review. J Surg Oncol 2019;: 1–10.

[4] Brandwein-Gensler M, Urken M, Wang B. Collision tumor of the thyroid: a case report of metastatic liposarcoma plus papillary thyroid carcinoma. Head Neck 2004; 26: 637–41. doi: 10.1002/hed.20024 15229907

[5] qingchao mu, yunfu cui, ma mimi, yan tian. Two rare cases of ovarian collision tumor, 25. PREPRINT (Version 1) available at Research Square 2020;.

[6] Tan GC, Chandramaya SF, Noordin A, S Tay PY. Collision tumor of the ovary: fibroma and mature cystic teratoma. Indian J Pathol Microbiol 2021; 64: 171–3. doi: 10.4103/IJPM.IJPM_670_19 33433434

[7] Peng Y, Lin J, Guan J, Chen L, Zhang X, Li S, et al. Ovarian collision tumors: imaging findings, pathological characteristics, diagnosis, and differential diagnosis. Abdom Radiol 2018; 43: 2156–68. doi: 10.1007/s00261-017-1419-6 29198011

[8] Troiano RN, Lazzarini KM, Scoutt LM, Lange RC, Flynn SD, McCarthy S. Fibroma and fibrothecoma of the ovary: MR imaging findings. Radiology 1997; 204: 795–8. doi: 10.1148/radiology.204.3.9280262 9280262

[9] Shinagare AB, Meylaerts LJ, Laury AR, Mortele KJ. Mri features of ovarian fibroma and fibrothecoma with histopathologic correlation. AJR Am J Roentgenol 2012; 198: W296–303. doi: 10.2214/AJR.11.7221 22358029

[10] Ting Y, Yang LI, Juan Z, Xing W, Feng YX. Ovarian thecoma with massive pleural effusion in postmenopausal women: a case report. Mol Clin Oncol 2016; 4: 1003–5. doi: 10.3892/mco.2016.853 27284435 PMC4887833

[11] AlGhamdi M, AlMutairi B, AlOsaimi A, Felemban A, AlYahya M. Mature cystic ovarian teratoma without intracystic fat: Case report with the "fat within the wall" sign. Radiol Case Rep 2020; 15: 367–70. doi: 10.1016/j.radcr.2020.01.011 32055261 PMC7005499

[12] Maddipati SS, P. SC, K. S, Sudha CP, Sowmya K. Collision tumor of ovary: a case report on bilateral dermoid cyst with co-existing unilateral mucinous cystadenoma. Int J Reprod Contracept Obstet Gynecol 2020; 9: 3078–80. doi: 10.18203/2320-1770.ijrcog20202764

[13] Park SB, Kim JK, Kim K-R, Cho K-S. Imaging findings of complications and unusual manifestations of ovarian teratomas. Radiographics 2008; ; 28: 969–83 Jul-Aug. doi: 10.1148/rg.284075069 18635624

